# Grain boundary oxidation of proton-irradiated nuclear grade stainless steel in simulated primary water of pressurized water reactor

**DOI:** 10.1038/s41598-020-80600-x

**Published:** 2021-01-14

**Authors:** Ping Deng, Qunjia Peng, En-Hou Han

**Affiliations:** 1 Nuclear Power Institute of China, Chengdu City, 610213 China; 2 Key Laboratory of Nuclear Materials and Safety Assessment, Institute of Metal Research, Chinese Academy of Sciences, Shenyang City, 110016 China; 3 Suzhou Nuclear Power Research Institute, Suzhou City, 215004 China

**Keywords:** Techniques and instrumentation, Structural materials

## Abstract

Grain boundary (GB) oxidation of proton-irradiated 304 nuclear grade stainless steel in primary water of pressurized water reactor was investigated. The investigation was conducted by studying microstructure of the oxide and oxide precursor formed at GB on an "atom-by-atom" basis by a combination of atom-probe tomography and transmission electron microscope. The results revealed that increasing irradiation dose promoted the GB oxidation, in correspondence with a different oxide and oxide precursor formed at the GB. Correlation of the oxide and oxide precursor with the GB oxidation behavior has been discussed in detail.

## Introduction

Irradiation-assisted stress corrosion cracking (IASCC) is a potential cause of material degradation that may lead to failure of austenitic stainless steel (SS) used in light water reactor core components. Up to date, numerous studies have been conducted to clarify the mechanism of IASCC^1–8^. It is widely accepted that localized deformation promoted by the irradiation in the alloy is a dominant mechanism for IASCC^2,4–8^. The irradiation produces high density of defect clusters such as dislocation loops and precipitates in the irradiated steels, which act as obstacles for dislocation motion and result in a heterogeneous localized deformation. The localized deformation is usually accompanied by significant changes in mechanical properties such as a large increase in yield strength and decrease in ductility^8–10^. Such changes in the irradiated steel promote localized strain accumulation at grain boundary (GB), which may result in nucleation sites of intergranular cracking^5,11,12^. The localized deformation, on the other hand, leads to formation of dislocation channels by annihilating the radiation-induced defects to GB^2,5,10,13^. As a result, the dislocation channeling could also be a potential contributor to IASCC by promoting dislocation pile-ups at the GB region and localized GB sliding. These investigations revealed that the changes in deformation modes by radiation induced microstructure and the interaction of localized dislocation channels with the GB promote the IASCC.

In the process of stress corrosion cracking, corrosion occurs simultaneously with localized deformation in the vicinity of the crack tip^14–16^, indicating that corrosion is one of the other potential contributors to IASCC. In fact, importance of the corrosion role in clarifying the mechanism of IASCC has been acknowledged. For instance, the irradiated stainless steels that are resistant to intergranular cracking at 288 °C in argon gas are susceptible to intergranular cracking in high temperature water at the same temperature^2^. This suggests that to fully clarify the IASCC mechanism, attentions should also be paid on the corrosion. In order to clarify the role of corrosion in IASCC, it at first needs to know how the irradiation affects the corrosion of the material, especially for the intergranular corrosion since the IASCC is usually in the intergranular mode and the initiation of intergranular SCC is directly related to the preferential intergranular corrosion^7,17,18^. For this reason, GB oxidation of the irradiated stainless steel will be investigated at nanoscale to attain a better understanding of the corrosion role in IASCC mechanisms.

The intergranular corrosion traditionally requires that a protective surface oxide film is not created^18–20^, which allows for the penetration of oxidation through the surface oxide film to the GB, while the subsurface GB oxidation is further dependent on protectability of intergranular oxide itself and the mass diffusion at the oxide front^21,22^. This suggests that to fully clarify the GB oxidation of the irradiated steel, attentions should be paid on how the irradiation affects the protectability of the surface and GB oxide, as well as the mass diffusion at the oxide front.

In recent years, the effect of irradiation on corrosion of austenitic SS has received increased attentions, while only a few studies have been reported. Our previous study as well as the study by Perrin et al. found an increase in the number and size of oxide particles formed on irradiated specimens, indicating a less protective surface oxide film by irradiation^23,24^. Promoted intergranular corrosion by irradiation was also confirmed in one study on irradiated 304 SS^23^. Discussion has mainly focused on the change in oxide composition by radiation-induce segregation (RIS). The possible change in oxide precursors and their effect on the GB corrosion, however, has not been studied. Since growth of the oxide is related to mass diffusion at the oxide/metal interface^22^, the oxide precursors and their interaction with microstructural defects at the oxide/matrix interface should play a role in the oxidation behavior.

The objective of this study was to present a more extensive understanding of the irradiation effects on GB oxidation in austenitic SS, using the atom probe tomography (APT) in conjunction with transmission electron microscope (TEM). Correlation of the oxide microstructure to oxidation along GB was discussed.

## Experimental

### Material and specimens

The material used for the experiment was solution annealed 304 nuclear grade SS (304NG SS), and alloying constituents was reported in a previous study^23^. Corrosion specimens with a dimension of 20 mm × 3 mm × 2 mm were cut from the steel, followed by wet grounding using SiC papers to a final grit of 3000# and diamond pastes polishing to achieve mirror-like surfaces. At last, the specimens were exposure to 40-nm colloidal silica slurry polishing for about 2 h to ensure the removal of surface residual strain before proton irradiation. The irradiation was conducted using 2 meV protons to a dose of 0.5 and 3 displacement per atom (dpa) at a dose rate of about 6 × 10^–6^ dpa per second (dpa/s) in a Tandetron accelerator at Michigan Ion Beam Laboratory. The irradiation temperature was controlled at 360 °C, monitored by a two-dimensional thermal imager, which could keep the target temperature variation within ± 10 °C during the irradiation. As presented in a previous work^23^, the irradiation created a nearly uniform damage profile within the ~ 1 to ~ 10 μm of the whole 20 µm-thick damaged layer, and showed an implantation peak at ~ 18 μm. Additional details of the irradiation setup and procedure have been available elsewhere^25,26^.

### Analysis of RIS at grain boundary

Following the irradiation, characterization of RIS at GB on 0.5- and 3-dpa irradiated specimens was performed in an APT system (CAMECA LEAP 4000X HR). Samples for the APT examination were prepared by a FEI QUANTA 200 3D focus ion beam (FIB) system, which greatly increased the chance of hitting a selected GB in an APT tip. The random high-angle grain boundaries were of interest to select, while the small-angle and twin grain boundaries were avoided by using ion channeling contrast of the grains. The ion channel contrast corresponded to the secondary electron emission coefficient, which varied as a function of the crystallographic orientation. As a result, the grains showed different contrasts depending on their orientations. The grains sharing a small-angle boundary usually have similar ion channeling contrast due to their small orientation difference. The high-angle and twin grain boundaries, on the other hand, generally showed visible ion channeling contrast, which was used to reveal the location of the boundaries. In addition, the high-angle boundaries were usually curved, while twin boundaries were generally straight in appearance. This further aided in the boundary identification. Once a high-angle GB was selected, the APT tips were prepared using the traditional lift-out techniques, by which sample wedges were cut from the selected GB, welded on to Si micro-posts and then annular milled to diameters of ~ 100 nm. Details of the lift-out techniques were described elsewhere^27^. The milled tips were subsequently analyzed in the APT system at a base temperature of 50 K in laser pulsing mode with an optimized laser energy of 60 pJ and a pulse repetition frequency of 200 kHz, which allowed a quicker evaporation of the tip and minimized chances of fracture failing. Following the evaporation, the commercial CAMECA Imago Visualization and Analysis Software package (IVAS, Version 3.6.8) was used to reconstruct and analyze the data.

### Corrosion test in simulated primary water

Prior to corrosion test, the irradiated specimens were once again polished by 40-nm colloidal silica slurry for about an hour, in order to achieve consistent surfaces of the 0.5- and 3-dpa irradiated specimens by removing the surfaces with a thickness of 1–2 μm. Then exposure of the specimens in simulated primary pressurized water reactor (PWR) water was conducted in a refreshed 316L autoclave at 320 °C for a fixed period of 500 h. The primary water in the autoclave contained 1200 mg/L of B as H_3_BO_3_ and 2.3 mg/L of Li as LiOH·H_2_O, and refreshed at a low flow rate of roughly 100 mL/min at 13 MPa pressure. Before ramping the autoclave to target temperature, the water was deoxygenated to ≤ 5 ppb dissolved oxygen by bubbling H_2_ gas into the water tank, followed by storing H_2_ in the tank with a hydrogen overpressure of 0.08 MPa, resulting in a dissolved hydrogen concentration of 2.6 mg/L. After the exposure, the specimens were removed from the autoclave for characterization of the oxide scale.

### Analysis of oxide at grain boundary

Following the corrosion test, characterization of the oxide scale formed at grain boundaries in the 0.5- and 3-dpa irradiated specimens was performed to clarify the GB oxidation behavior, including the cross-section observation by a JEM-2100 TEM and the composition characterization by APT. The TEM was equipped with a Gatan 4k charge coupled device camera for capturing high-resolution TEM images. The high-resolution TEM images were involved for identifying the oxides at the GB as well as the metallic grain boundaries ahead of the oxidation front. The accurate composition profile of the oxide and oxide precursors, on the other hand, was analyzed by APT equipped with a reflection lens for high mass resolution. Additional descriptions of the APT procedure and parameters were previously presented in the section of RIS analysis.

Both the TEM and APT samples were prepared using the FEI QUANTA 200 3D FIB system, so that precise sampling of the cross-section of the oxide scale containing a portion of the same selected GB for the TEM observation and APT analysis can be attained. To be consistent with the RIS analysis, only the random high-angle GB was selected to analyze the intergranular corrosion behavior, and sampling intervals of the TEM and APT samples were controlled at 1–2 μm. One TEM sample was produced from the selected GB and a series of APT samples were extracted from the lifted-out wedge following the method described in “Analysis of RIS at grain boundary” section.

## Results

### RIS at grain boundary in 304NG SS

**Figure 1:**
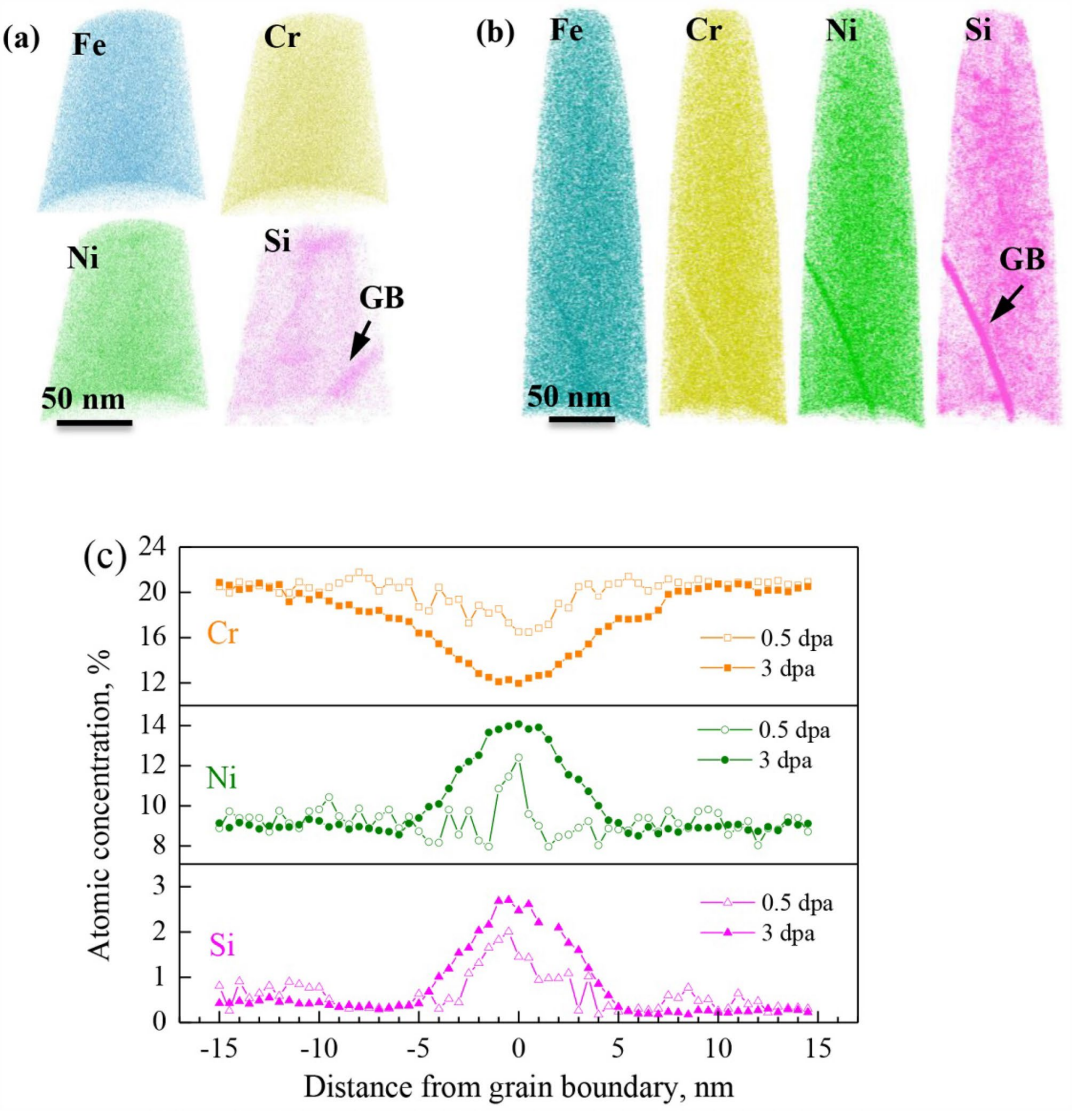
APT characterizations of 304NG SS following proton irradiation to a dose of 0.5 and 3 dpa at 360 °C. (**a**) and (**b**) Atom maps of a reconstructed volume from a data set of the 0.5- and 3-dpa irradiated steel, showing depletion of Cr and Mn and enrichment of Fe, Ni and Si at grain boundary. The position of grain boundary is highlighted by the arrow. (**c**) 1D concentration profiles across the grain boundary in the analyzed volume. *Atom maps in (**a**) and (**b**) were reconstructed using the commercial CAMECA Imago Visualization and Analysis Software package (IVAS, Version 3.6.8). URL link: https://www.cameca.com/.

Color-coded APT atom maps of the specimens following the irradiation to a dose of 0.5 and 3 dpa at 360 °C are shown in Fig. 1a,b. As illustrated, an enrichment of Ni, Si and Fe, and a depletion of Cr at GB were evident from the individual atom density map on both specimens. While a similar nature of RIS was observed on the 0.5- and 3-dpa irradiated specimens, the magnitude of RIS showed difference, as displayed by the composition profiles across the grain boundaries shown in Fig. 1c The average RIS magnitude at the GB on 0.5-dpa irradiated specimen was measured to be 3.1 ± 0.3, 3.2 ± 0.2 and 1.8 ± 0.4 at% for Cr, Ni and Si, respectively, and increased to 7.8 ± 0.4, 5.2 ± 0.5 and 2.8 ± 0.3 at% by the 3-dpa irradiation, which suggested a higher magnitude of RIS at GB by increasing the irradiation dose. Figure 1e,f summarize the magnitude of RIS at the two grain boundaries.

### Oxide at grain boundary observed by TEM

**Figure 2:**
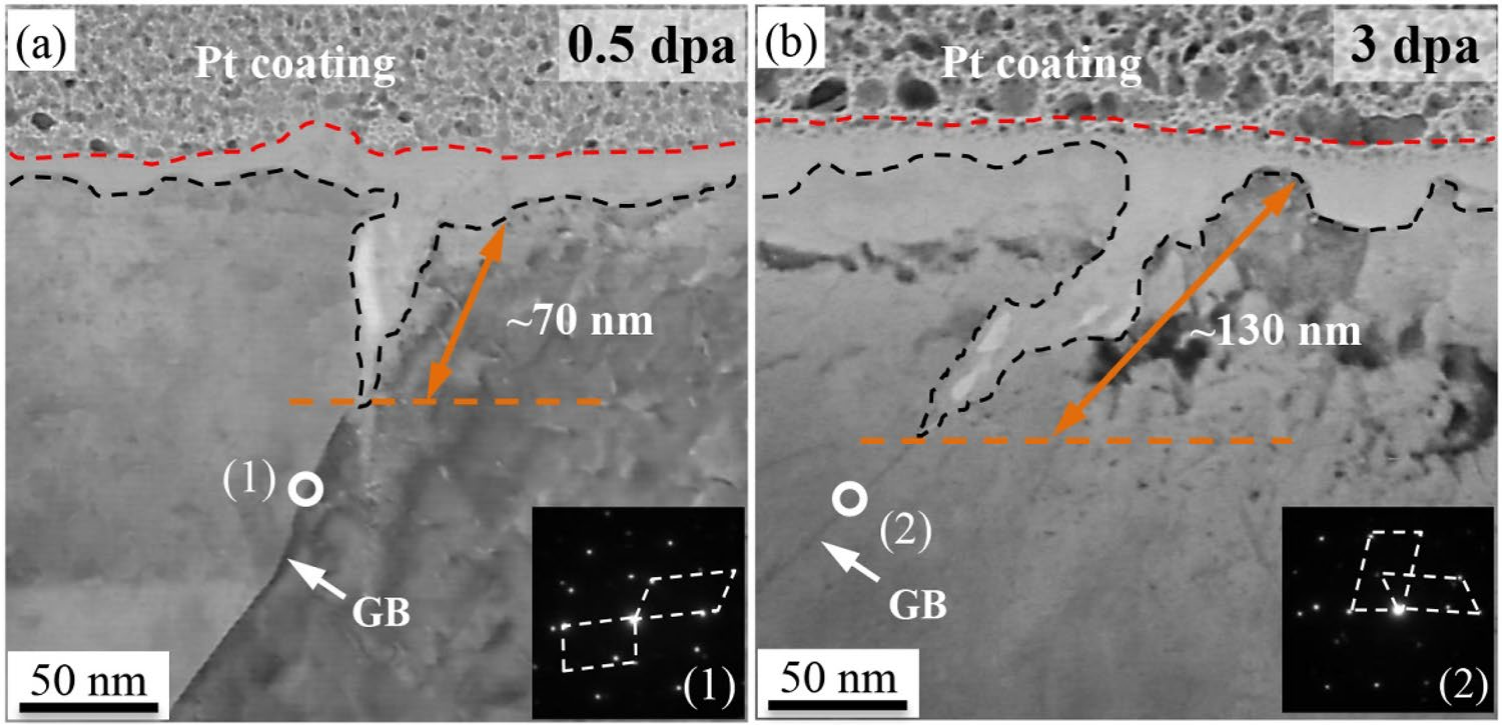
TEM observation of the oxide at the grain boundary in (**a**) 0.5- and (**b**) 3-dpa irradiated 304NG SS.

Figure 2 shows the TEM observation of the morphology of the oxide formed at the GB in 0.5- and 3-dpa irradiated specimens. It was found that a sharp oxide was formed at the grain boundary, suggesting a typical nature of intergranular corrosion. While the morphology of the oxide in 0.5- and 3-dpa irradiated specimens was similar, the depth of the penetration oxidation through the surface oxide film into the grain boundary differed with the irradiation dose. As shown in Fig. 2a,b, a penetration of oxidation with a depth of approximately 70 nm was observed on the 0.5-dpa specimen, and it increased to ~ 130 nm by increasing the irradiation dose to 3 dpa. This suggested a promoted intergranular corrosion by the irradiation.

Composition profiles of the oxide formed at the GB in 0.5- and 3-dpa irradiated specimens were performed by APT, as to be stated in the following section.

### Oxide at grain boundary analyzed by APT

**Figure 3:**
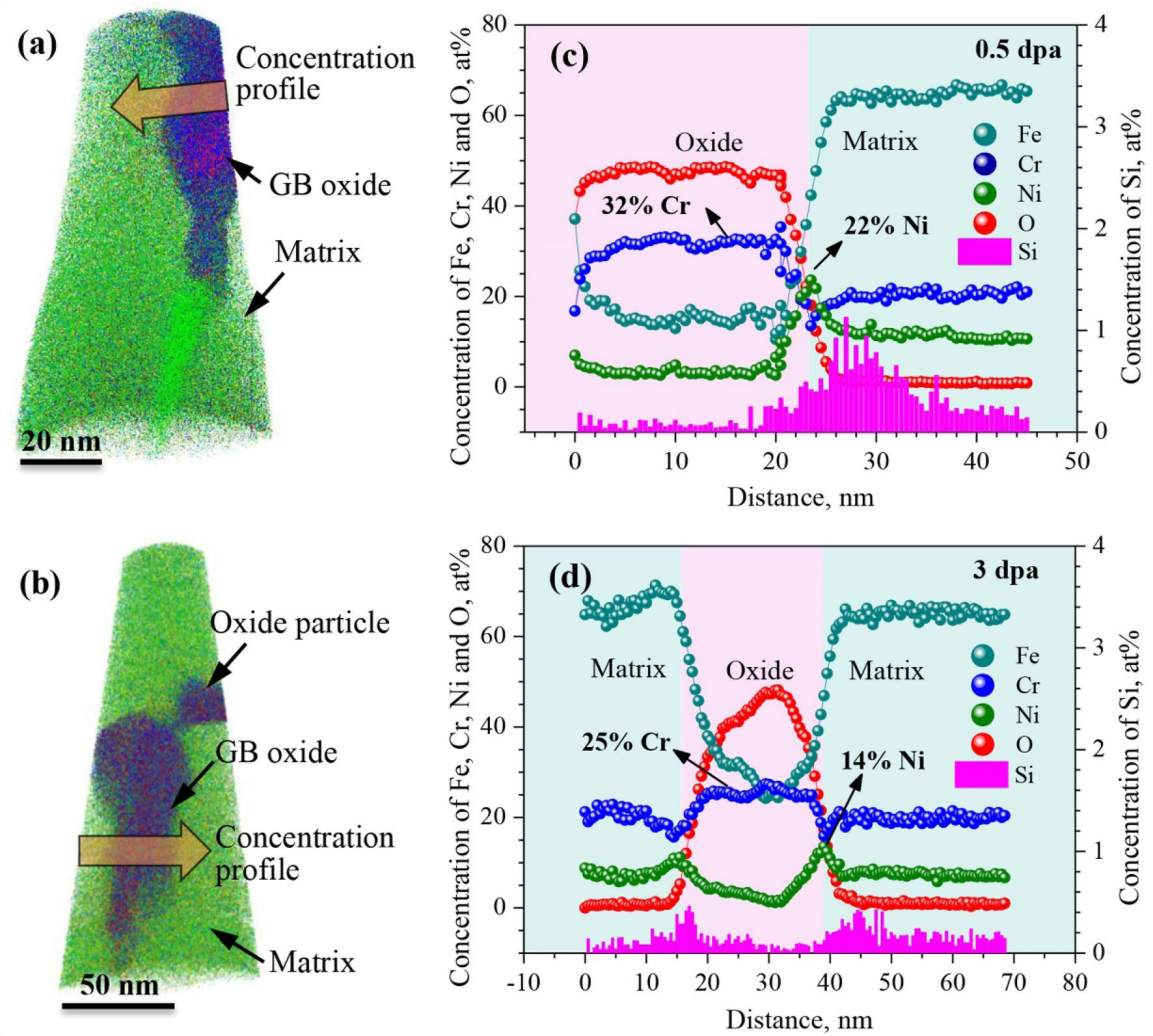
(**a**) and (**b**) Atom density maps of the 0.5- and 3-dpa irradiated specimens following the 500-h exposure in primary water at 320 °C, showing oxidation along the grain boundaries. (**c**) and (**d**) Corresponding 1D concentration profiles across the oxide/matrix interfaces, showing the oxide to be Cr-rich with asymmetric Ni and Si enrichment at the oxide/matrix interfaces. *Atom maps in Fig. 2a,b were reconstructed using the commercial CAMECA Imago Visualization and Analysis Software package (IVAS, Version 3.6.8). URL link: https://www.cameca.com/.

Figure 3a,b show two APT data sets containing part of the same grain boundaries in the 0.5- and 3-dpa irradiated specimens that were characterized in the TEM. Both data sets consist of two regions with different compositions, i.e. an oxidized GB going almost vertically down the matrix and the matrix at the bottom. The concentration profiles across the oxide/matrix interfaces shown in Fig. 3c,d revealed the composition of the oxide formed at the GB in the 0.5- and 3-dpa irradiated specimens. While the oxides were both depleted in Fe, Ni, Si and enriched in Cr, the Cr content varied with the irradiation dose. It was 32 at% in the oxide in the 0.5-dpa irradiated specimen, and decreased to 25 at% by increasing the irradiation to 3 dpa. Further, the profiles in Fig. 3c,d also displayed the composition changes across the oxide/matrix interface layer. A similar thin Ni- and Si-rich interface layer separating the matrix and oxide regions was observed in both the 0.5- and 3-dpa irradiated specimens. In the interface layers, the Ni and Si concentration in the interface layer in the 0.5-dpa irradiated specimen peaked at about 25 and 1 at%, respectively, and decreased to ~ 14 and 0.5 at% by increasing the irradiation to 3 dpa. This suggested a decreased enrichment of Ni and Si ahead of the interface between the oxide and the matrix by a higher irradiation dose.

**Figure 4:**
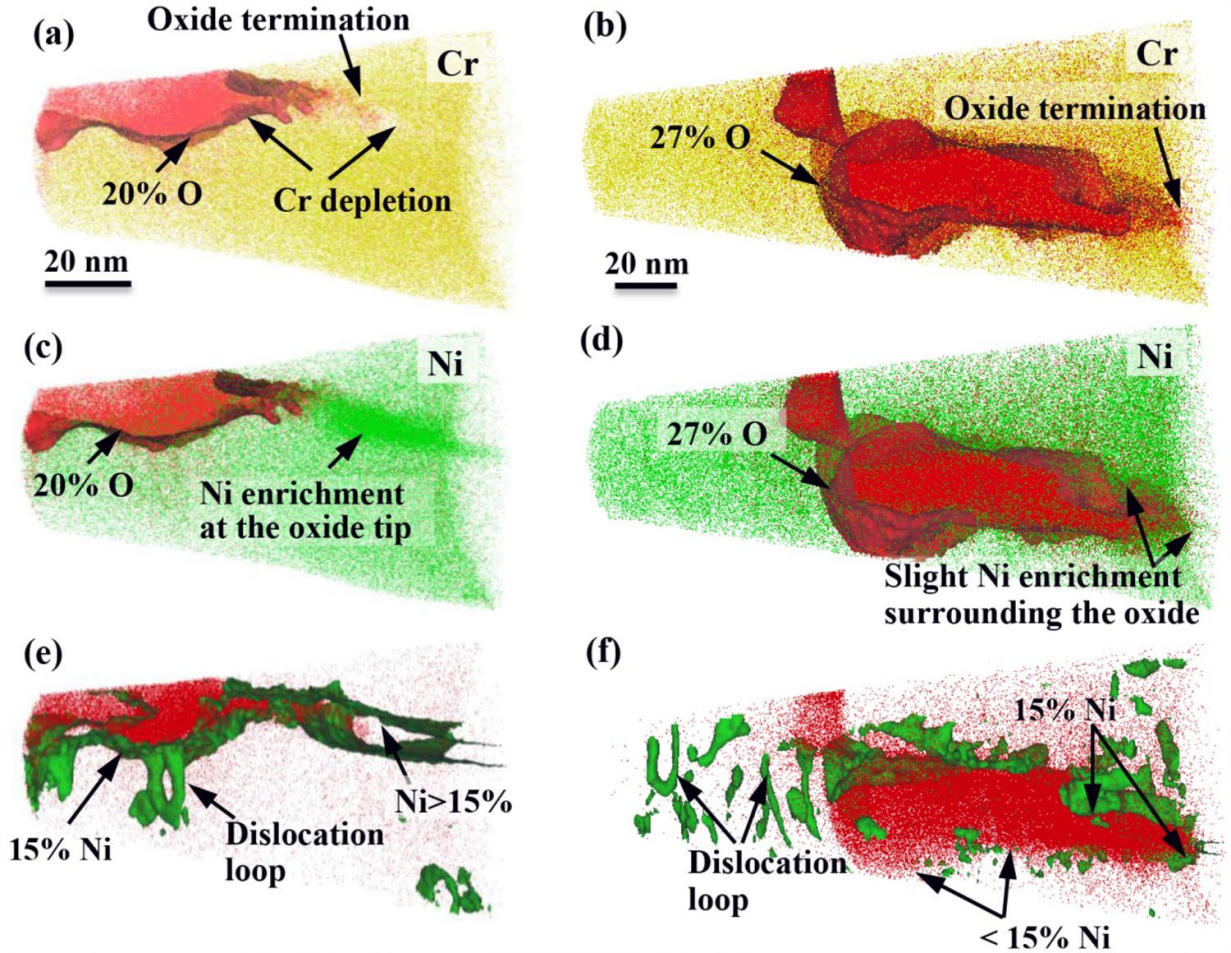
APT analysis of element distributions at the grain boundary oxide/matrix interfaces on the 0.5- and 3-dpa irradiated specimens after exposure for 500 h in primary water at 320 °C. (**a**) and (**b**) Cr distributions ahead of the oxides. (**c**) and (**d**) Ni distributions ahead of the oxides. (**e**) and (**f**) Iso-concentration surface plot of Ni at 15% (green). A localized Ni enrichment with a concentration of 67 at% ahead of the oxide was observed on the 0.5-dpa irradiated specimen, while the 3-dpa irradiated specimen showed a slight enrichment of Ni ahead of the whole interface between the oxide and the matrix. *Element distributions in this figure were reconstructed using the commercial CAMECA Imago Visualization and Analysis Software package (IVAS, Version 3.6.8). URL link: https://www.cameca.com/.

More details of the elements distribution at the oxide/matrix interfaces was observed and analyzed by using the iso-concentration surface analysis technique (Fig. 4). The iso-concentration surface shows regions containing more/less than a given concentration of a particular element, which aids in elements distribution analysis at the interface. A concentration of 20 and 27% oxygen was employed for the 0.5- and 3-dpa irradiated specimens to reveal location of the oxide/matrix interfaces (Fig. 4a–d). At the interfaces, difference in the Ni-enrichment behavior adjacent to the oxide was observed. While both specimens showed a slight Ni enrichment ahead of the whole interface between the GB oxide and the base metal, a localized enrichment of Ni with a concentration of ~ 67 at% was also observed ahead the tip of the GB oxide in the 0.5-dpa specimen (Fig. 4c,d). This was further confirmed in Fig. 4e,f by employing iso-concentration surface of Ni with a concentration of 15 at%, which was much higher than that in the base metal (8.87 at%). The iso-concentration surface at 15 at% Ni in the 0.5-dpa specimen gave rise to a connected volume with an overall high Ni content, while the 3-dpa irradiated specimen showed a discrete concentration plot of Ni surrounding the oxide surface, suggesting a slight enrichment of Ni ahead of the interface. This was in agreement with the concentration profiles across the oxide/matrix interfaces shown in Fig. 3c,d.

**Figure 5:**
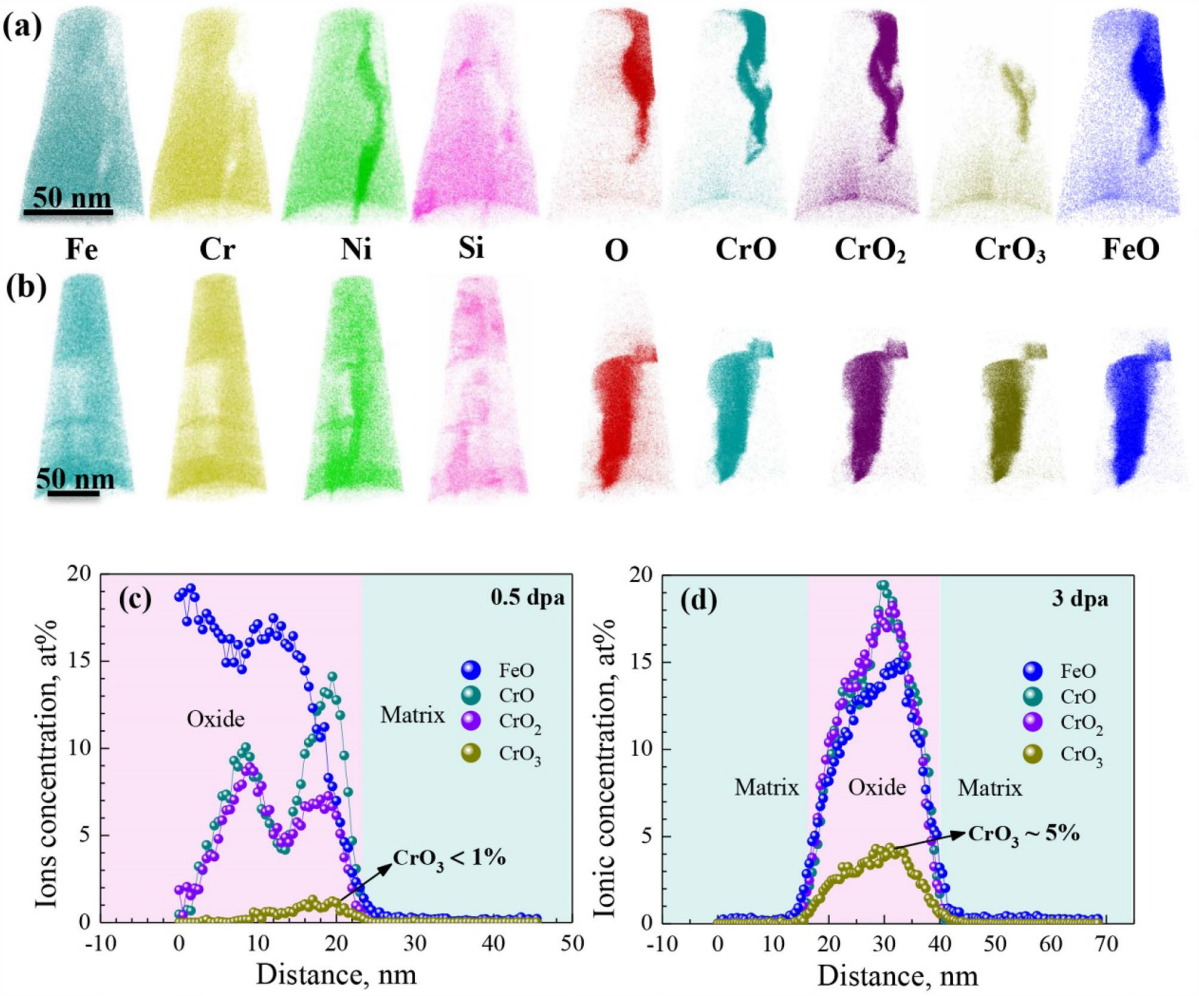
(**a**) and (**b**) Atom density maps showing the evaporated species for the grain boundary oxides on the 0.5- and 3-dpa irradiated specimens. The orientation of both data sets is identical to the orientation in Fig. 2a,b, where all ions of these data sets are shown in the same map. (**c**) and (**d**) Corresponding ions concentration profiles across the grain boundary oxides. *Atom density maps in this figure were reconstructed using the commercial CAMECA Imago Visualization and Analysis Software package (IVAS, Version 3.6.8). URL link: https://www.cameca.com/.

Figure 5a,b show the separate maps of the evaporated species for the oxides formed at the grain boundaries in the 0.5- and 3-dpa specimens. It revealed a similar ion types prevalent in different parts of both oxides. A continuous oxygen signal was observed throughout the entire oxides, where there was a decrease in the alloying species such as Fe, Cr, Ni and Si. Further, while a slight density of Ni and Si was present in the GB oxide, NiO_x_ and SiO_x_ were completely undetected in all oxidized regions, suggesting the possible absence of Ni and Si oxidization. On the other hand, the presence of CrO_3_ suggested that all the Cr in oxides was likely oxidized since CrO_3_ was formed subsequently to CrO and CrO_2_ in case of additional oxygen. The plots of FeO, CrO and CrO_2_ corresponded well to the oxygen map, while the morphology of CrO_3_ varied with the irradiation dose. In the 3-dpa irradiated specimen, a widespread CrO_3_ was observed throughout the oxide. In contrast, the CrO_3_ in the 0.5-dpa irradiated specimen was not continuous but showed a type of filaments, suggesting a difference in concentration. Concentration profiles of evaporated species across the GB oxides in Fig. 5c,d revealed that concentration of CrO_3_ was increased by a higher irradiation dose (< 1% for 0.5-dpa and ~ 5% for 3-dpa).

## Discussion

The previous TEM and APT analysis shown in Figs. 3, 4 and 5 revealed that the oxide formed at the GB in the 3-dpa irradiated specimen had a lower Cr content but a higher concentration of CrO_3_ than that in the 0.5-dpa irradiated specimen, indicating that increasing the irradiation dose led to a difference in the microstructure of the oxide formed at the GB in 304NG SS in primary PWR water. This is primarily attributed to the depletion of Cr at the primary GB by RIS, as has been discussed in a previous study^23^. In addition, the depletion of Cr by RIS also accounts for the formation of CrO_3_ by decreasing the content ratio of Cr/O in the oxide at the GB.

The APT analysis in Figs. 3 and 4 also revealed Ni enrichment ahead of the interface between the oxide and the matrix was decreased by a higher irradiation dose. Further, a localized enrichment of Ni was also observed ahead the tip of the GB oxide in the 0.5-dpa specimen. The enrichment of Ni adjacent to the oxide most likely resulted from the selective oxidation of Fe and Cr^28–30^. According to the Gibbs free energy-temperature (ΔG° − T) map of Cr-, Fe-, and Ni-oxides, the critical oxygen partial pressure required for formation of the oxides is Cr < Fe < Ni^30^, indicating that the Cr- and Fe-oxides were formed prior to the Ni-oxides. This was also supported by the absence of evaporated NiO_x_ in the GB oxides (Fig. 5a,b). As the diffusion rate of Ni in Cr-enriched oxide is much lower than that of Fe^21,31^, the preferential oxidation of Cr along the GB could push Ni into the base metal by the growing Cr-oxide^29,32–35^. This led to the enrichment of Ni at the oxide/matrix interface. As such, the different Ni enrichment behavior adjacent to the oxide is again attributed to the difference in the composition of the primary GB in 0.5- and 3-dpa irradiated specimens. During the irradiation, RIS occurred as a result of the unequal participation of solutes in the vacancy and interstitial fluxes to sinks, while the depletion of Cr at GB was formed due to its faster diffusion rate than Fe and Ni^3,36^. According to the APT analysis of RIS shown in Fig. 1, increasing the irradiation dose led to a higher magnitude of Cr-depletion at GB. Consequently, the primary GB in 0.5-dpa irradiated specimen had a relative higher Cr content than that following 3-dpa irradiation. A higher content of Cr promoted more Cr-oxide formed at the GB, leading to a higher Ni enrichment adjacent to the oxide. Further, as GB is a more effective diffusion path than the grain^33,37^, localized Ni enrichment with a higher concentration ahead of the oxide tip can also be expected.

As has been acknowledged, Cr content in an oxide scale formed on austenitic alloys in high temperature water could affect the protectability of the oxide^18,38,39^. As such, the different oxidation behavior along the GB in 0.5- and 3-dpa irradiated specimens resulted from a combined effect of the change in Cr content of the intergranular oxide and Ni enrichment adjacent to the oxide. A higher Cr content in oxide indicated a higher protectability of the oxide, which could mitigate further oxidation by decreasing oxygen diffusion rate through the oxide itself^18,23,38,39^. As a result, a lower GB oxidation rate was observed in the 0.5-dpa irradiated specimen (Fig. 3) due to the lower Cr content in oxide by RIS^23^.

On the other hand, the depletion of Cr by RIS also affected the Ni enrichment behavior adjacent to the oxide. Difference in the Ni enrichment behavior led to difference in further oxidation along GB. The APT data in Fig. 4 shows enhanced oxidation just above the Ni-rich particle. From this it appears probable that Ni enrichment can stop or at least delay further oxygen diffusion along the GB. As a result, the lower GB oxidation rate on the 0.5-dpa specimen is a combined effect of a higher protectability of the oxide itself and a smaller diffusion rate of oxygen at the oxide/matrix interface.

**Figure 6:**
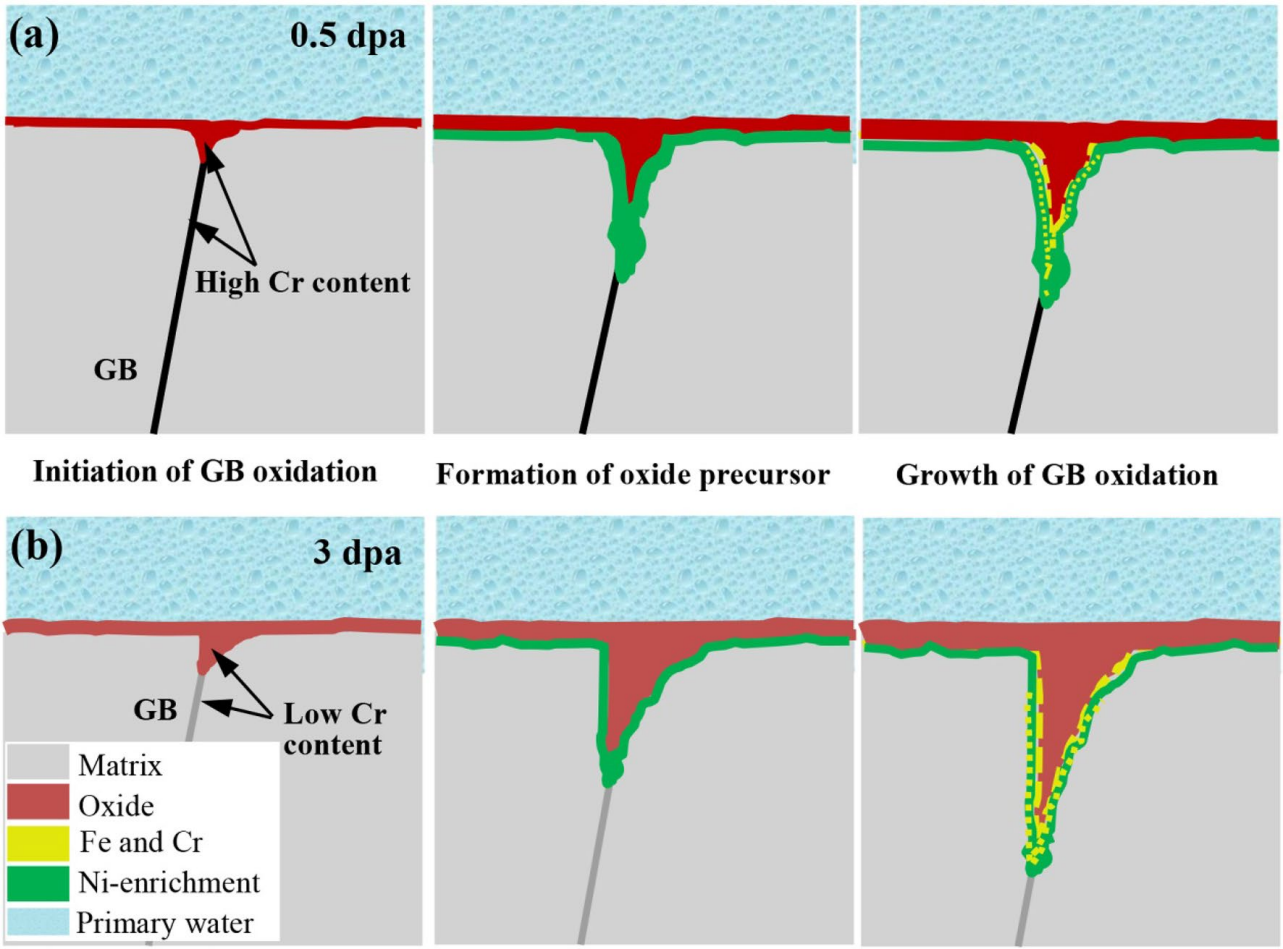
Schematics showing the effect of oxide microstructure on grain boundary oxidation on the (**a**) 0.5- and (**b**) 3-dpa irradiated specimens in primary water at 320 °C. *Schematics in this figure were made using the PPT Software package (Office, Version 2010).

The GB oxidation process for the 0.5- and 3-dpa irradiated specimens as discussed above are schematically shown in Fig. 6.

## Conclusions

GB oxidation of proton-irradiated 304NG SS in primary PWR water was successfully investigated by a combination of APT and analytical TEM. The following conclusions can be drawn from the investigation:

1. GB segregation of Fe, Cr, Ni and Si by RIS is measured by APT, while the RIS was increased by a higher irradiation dose.
2. RIS decreases Cr content in the oxide formed at the GB in the steel in primary PWR water, in addition to the Ni enrichment adjacent to the oxide.
3. Increasing the irradiation dose led to a higher GB oxidation rate in primary PWR water. This is attributed to a decreased Cr content in the oxide and Ni enrichment adjacent to the oxide by RIS.
