# Author Correction: Grain boundary oxidation of proton-irradiated nuclear grade stainless steel in simulated primary water of pressurized water reactor

**DOI:** 10.1038/s41598-021-88868-3

**Published:** 2021-04-22

**Authors:** Ping Deng, Qunjia Peng, En-Hou Han

**Affiliations:** 1 Nuclear Power Institute of China, Chengdu City, 610213 China; 2 Key Laboratory of Nuclear Materials and Safety Assessment, Institute of Metal Research, Chinese Academy of Sciences, Shenyang City, 110016 China; 3 Suzhou Nuclear Power Research Institute, Suzhou City, 215004 China

Correction to: *Scientific Reports* 10.1038/s41598-020-80600-x, published online 14 January 2021

In the original version of this Article Qunjia Peng was incorrectly affiliated with ‘Nuclear Power Institute of China, Chengdu City, 610213, China’. The correct affiliations are listed below.

Key Laboratory of Nuclear Materials and Safety Assessment, Institute of Metal Research, Chinese Academy of Sciences, Shenyang City, 110016, China.

Suzhou Nuclear Power Research Institute, Suzhou City, 215004, China.

The original Article has been corrected.

